# A Rare Case of Extraosseous Ewing’s Sarcoma/Primitive Neuroectodermal Tumor in Female

**DOI:** 10.7759/cureus.72586

**Published:** 2024-10-28

**Authors:** K Mathenkumar, D Balaji, Harshwanth Chandhar

**Affiliations:** 1 General Surgery, Sri Ramaswamy Memorial (SRM) Institute of Science and Technology, Chengalpattu, IND

**Keywords:** ewing’s sarcoma, immunohistochemistry, primitive neuroectodermal tumor, small round cell, soft tissue

## Abstract

Extraosseous Ewing’s sarcoma (EES) is a rare form of Ewing's sarcoma that arises outside the bones in soft-tissue structures. It is thought to result from a genetic abnormality involving the fusion of the EWSR1 gene with various partner genes, most commonly the FLI-1 gene. Common symptoms include pain, swelling, and sometimes a palpable mass at the site of the tumor. Diagnosis typically involves imaging studies such as magnetic resonance imaging (MRI), computed tomography (CT) scans, and biopsy for confirmation. Treatment typically includes a combination of chemotherapy, surgery to remove the tumor, and sometimes radiation therapy.

In this report, we present a case of EES in a 44-year-old female who presented with complaints of swelling in the right thigh for 3 months. The mass was soft in consistency with well-delineated borders, evident both clinically and radiologically, extending into the subcutaneous layer and involving the right inguinal lymph nodes. The mass was widely excised with en-bloc dissection of the right inguinal nodes. The histopathological features confirmed the diagnosis and have been discussed in this study.

This case underscores the clinical significance of EES, a rare variant presenting diagnostic challenges. Our findings highlight the importance of prompt diagnosis and early management to improve outcomes for patients with this aggressive malignancy.

## Introduction

Ewing’s sarcoma (EWS), initially described by James Ewing in 1921, is a highly aggressive tumor affecting adolescents and young adults, comprising 10% to 15% of all bone sarcomas [1]. It encompasses classic bone Ewing sarcoma, extraosseous Ewing sarcoma (EES), Askin tumor (malignant small cell tumor of the chest wall), and soft-tissue-based primitive neuroectodermal tumors [2].

EES predominantly impacts young individuals and carries a poor prognosis, especially in metastatic cases, with high mortality rates [3]. It can manifest in various locations without specific clinical symptoms, which often leads to delayed diagnosis. Definitive diagnosis relies significantly on histopathological examination due to the absence of specific clinical and radiological indicators [2].

These sarcomas share similar histologic and immunohistochemical features, suggesting a common origin from mesenchymal progenitor cells [4]. Treatment typically involves surgical excision aimed at complete tumor removal while preserving organ function to the extent possible [2]. Despite historically high mortality rates, advancements in local therapy and multi-agent chemotherapy have substantially improved the 5-year survival rate, increasing it from less than 20% to over 70% [4].

## Case presentation

A 44-year-old female with no notable pathological history was admitted with complaints of swelling in the right thigh for the past 3 months. Initially, the swelling was small and gradually progressed to the present size of approximately 5×4 cm. It was initially painless; however, as the swelling progressed, it became associated with intermittent dull aching pain, with no aggravating or relieving factors reported. She denied any history of trauma, fever, discharge, or restriction of movements. Additionally, there was no history of weight loss, loss of appetite, breathing difficulties, or back pain.

**Figure 1:**
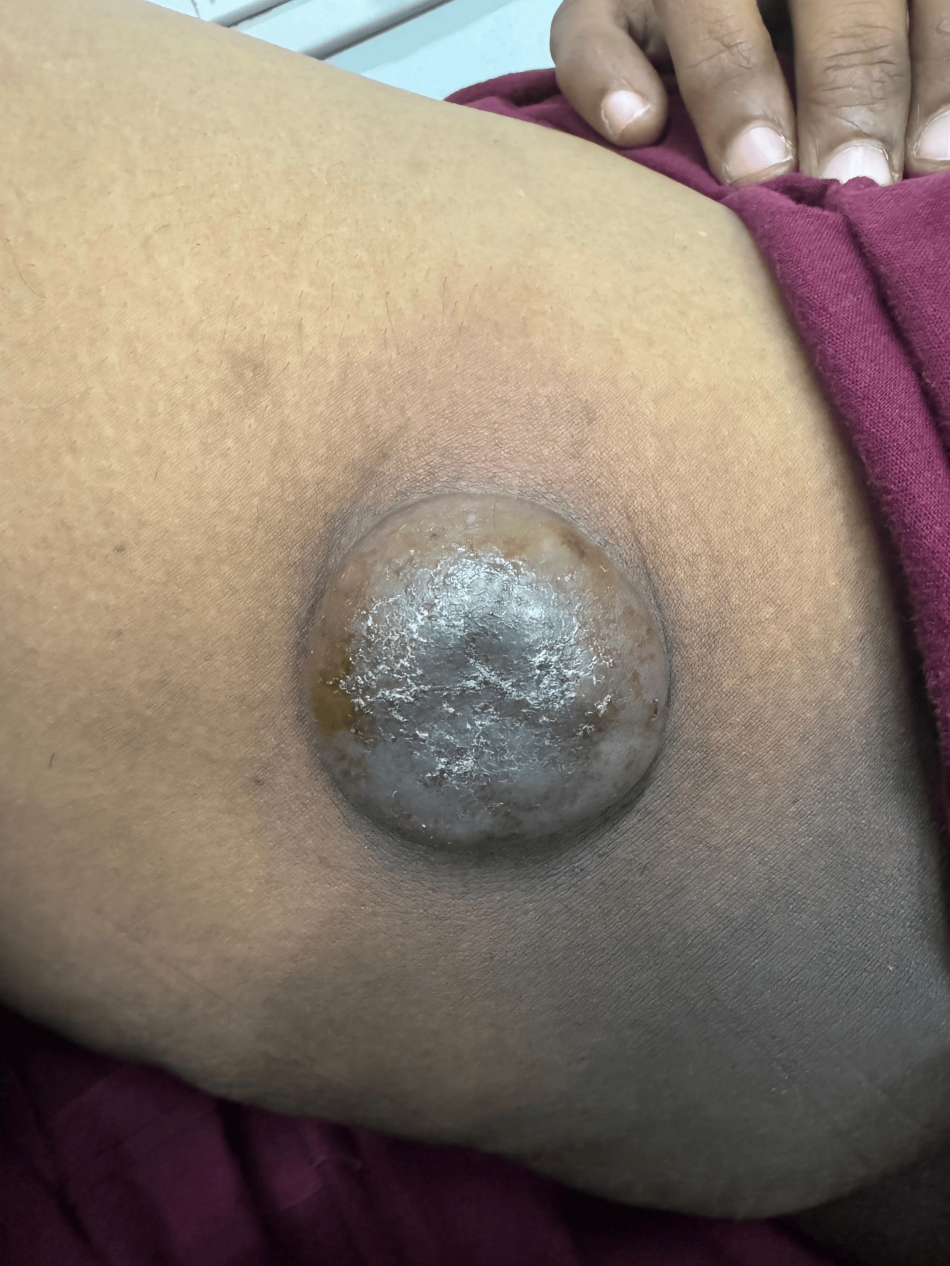
Anteroposterior view: Swelling in the medial aspect of the right proximal thigh

**Figure 2:**
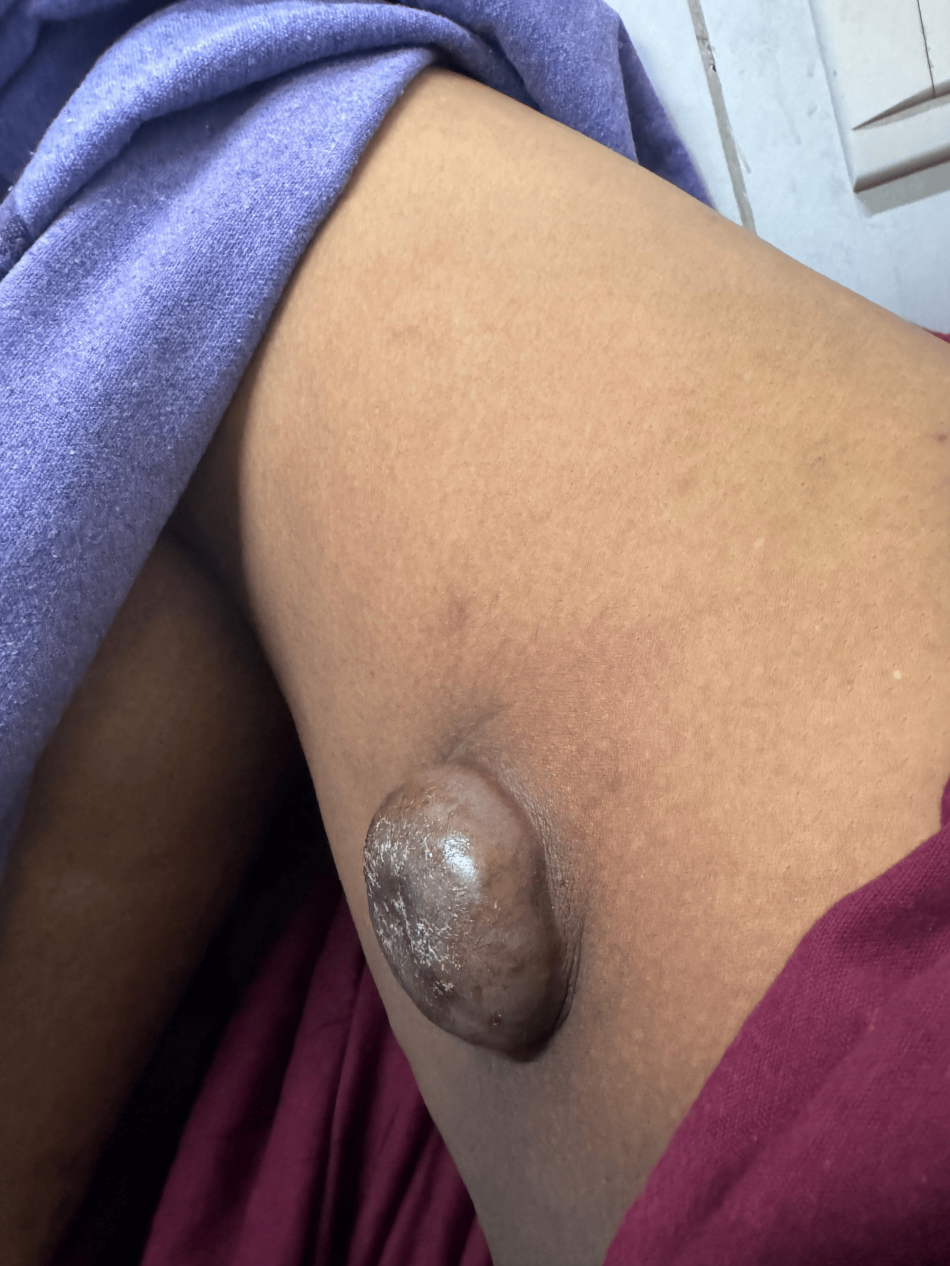
Lateral view: Swelling in the medial aspect of the right proximal thigh

The biochemical workup showed no abnormalities. Physical examination revealed a swelling approximately 5×4 cm in size located in the anteromedial aspect of the right thigh, around 8 cm below and lateral to the pubic tubercle and 10 cm below the inguinal crease line. It appeared globular in shape with a smooth surface. The skin over the swelling was hyperpigmented and non-pinchable, while the surrounding skin appeared normal (Figures 1-2). There was no local warmth or tenderness, and the swelling felt firm to hard in consistency with well-defined borders. The swelling was mobile in all directions and not fixed to the underlying muscle after contraction. It was fluctuant and irreducible, and no scar or sinus was observed over it. The ipsilateral inguinal group of lymph nodes was palpable.

**Figure 3:**
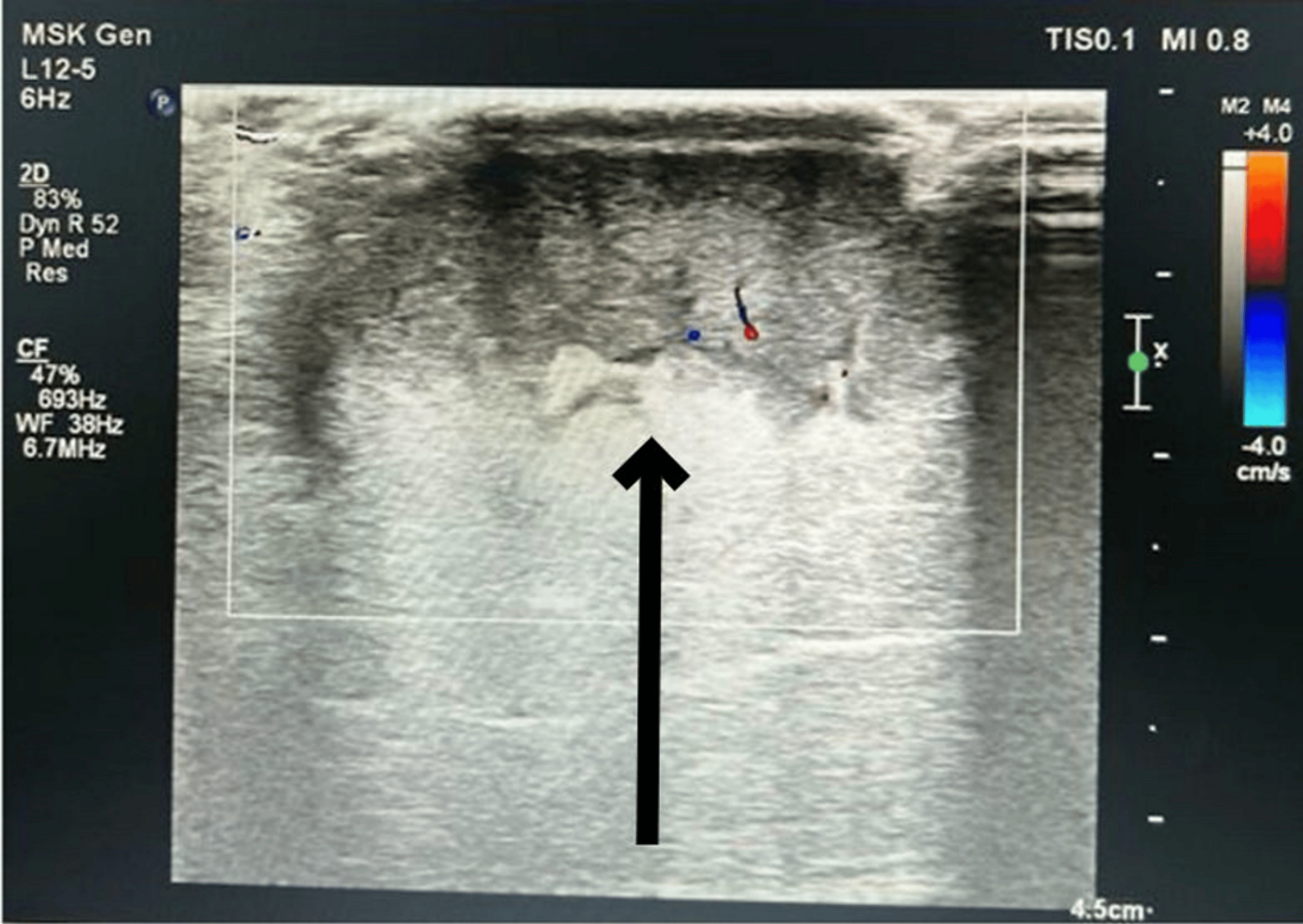
USG image: The soft-tissue swelling in the subcutaneous plane USG: ultrasonogram The black arrow shows soft-tissue swelling in the subcutaneous plane.

**Figure 4:**
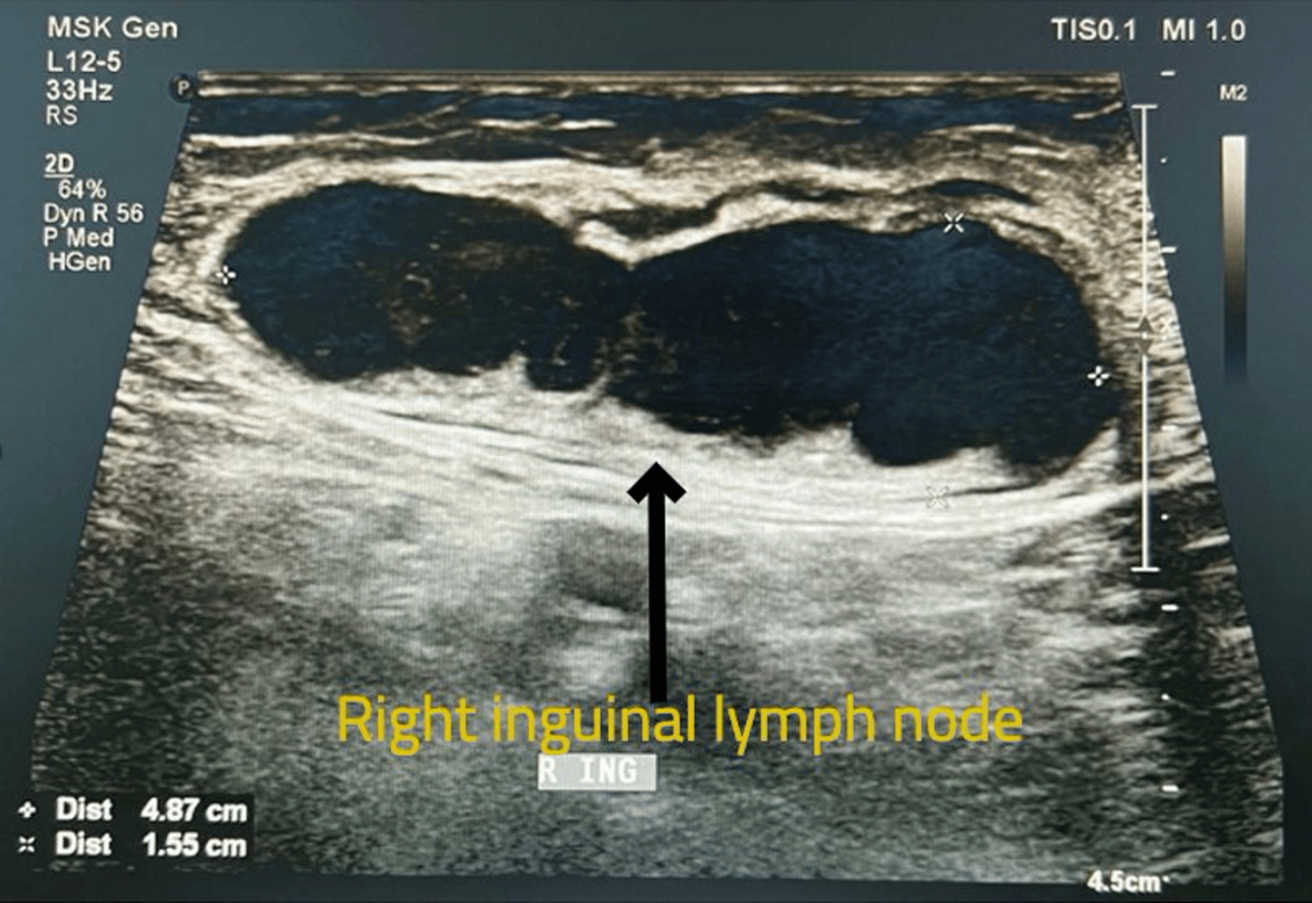
USG showing enlarged lymph nodes in the right inguinal region USG: ultrasonogram The black arrow shows an enlarged right inguinal lymph node.

**Figure 5:**
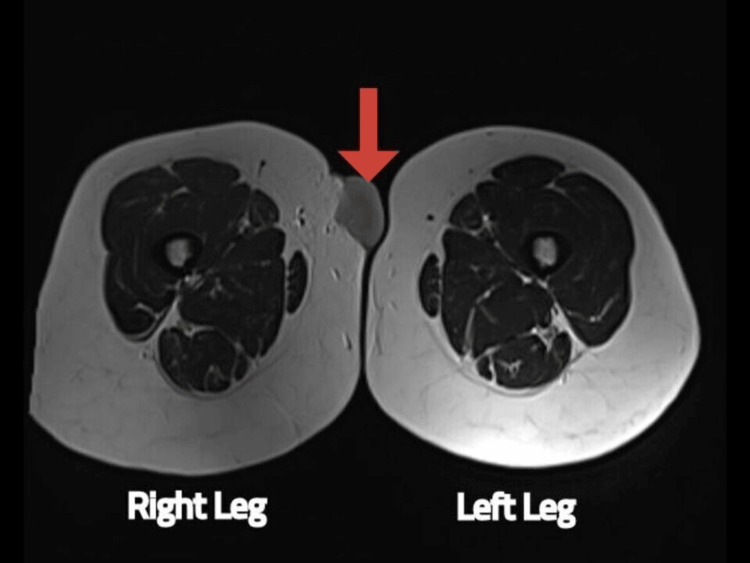
MRI of the proximal thigh shows soft-tissue swelling in the axial view MRI: magnetic resonance imaging The red arrow shows soft-tissue swelling in the right leg.

**Figure 6:**
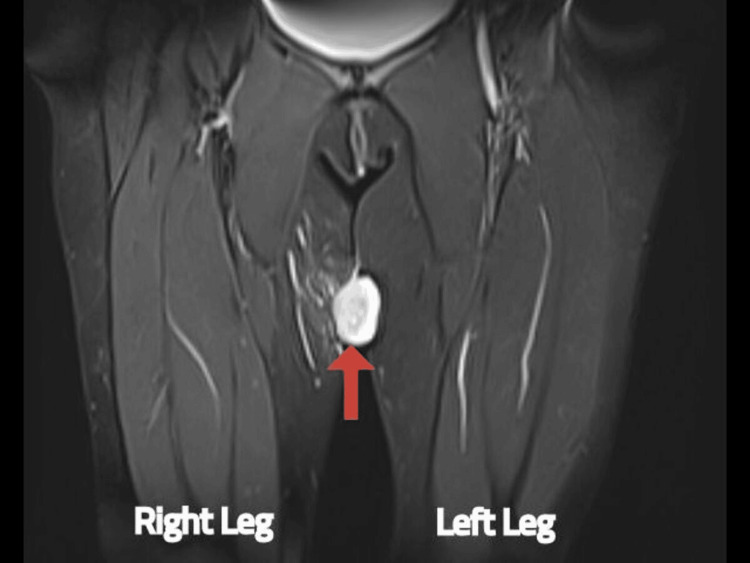
MRI of the proximal thigh shows soft-tissue swelling in the coronal view MRI: magnetic resonance imaging The red arrow shows soft-tissue swelling in the right proximal thigh.

Ultrasound examination of the right thigh showed a well-defined ovoid heterogeneous lesion in the skin and subcutaneous aspect of the upper third of the right thigh in its medial aspect with vascularity seen on doppler, enlarged lymph nodes with loss of fatty hilum in the right inguinal region (Figures 3-4). MRI right thigh revealed a well-defined short tau inversion recovery (STIR) hyperintense, T1 hypo intense T2 intermediate SI lesion measuring 3.8 × 3.0 cm noted involving the subcutaneous plane of anteromedial aspect of upper one-third of the right thigh with surrounding STIR hyperintensities (Figures 5-6). No obvious intramuscular or underlying bone extension was seen. Few tortuous vessels were seen adjacent to the lesion. Enlarged lymph nodes were noted in the right inguinal region, the largest measuring 4.6 × 1.7 cm. Magnetic resonance imaging (MRI) abdomen showed right inguinal lymph adenopathy and computed tomography (CT) chest was unremarkable.

**Figure 7:**
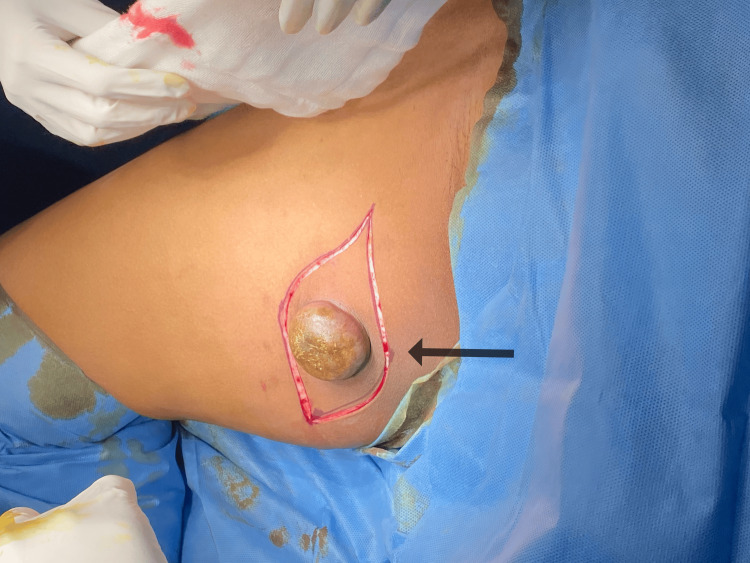
Intraoperative image showing wide elliptical incision The black arrow shows an elliptical incision around the swelling.

**Figure 8:**
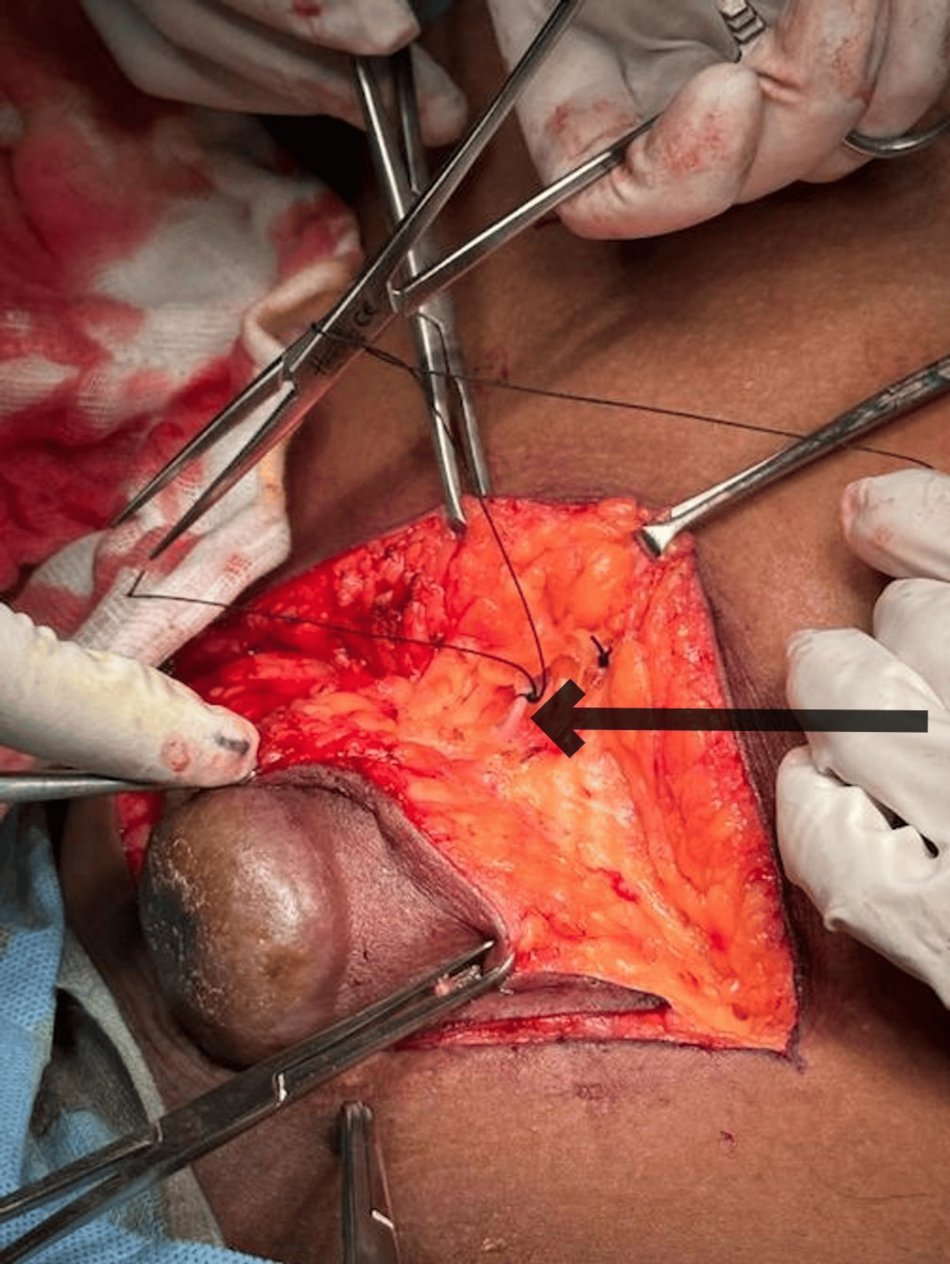
Intraoperative image showing ligation of GSV GSV: great saphenous vein The black arrow shows the ligation of the great saphenous vein.

**Figure 9:**
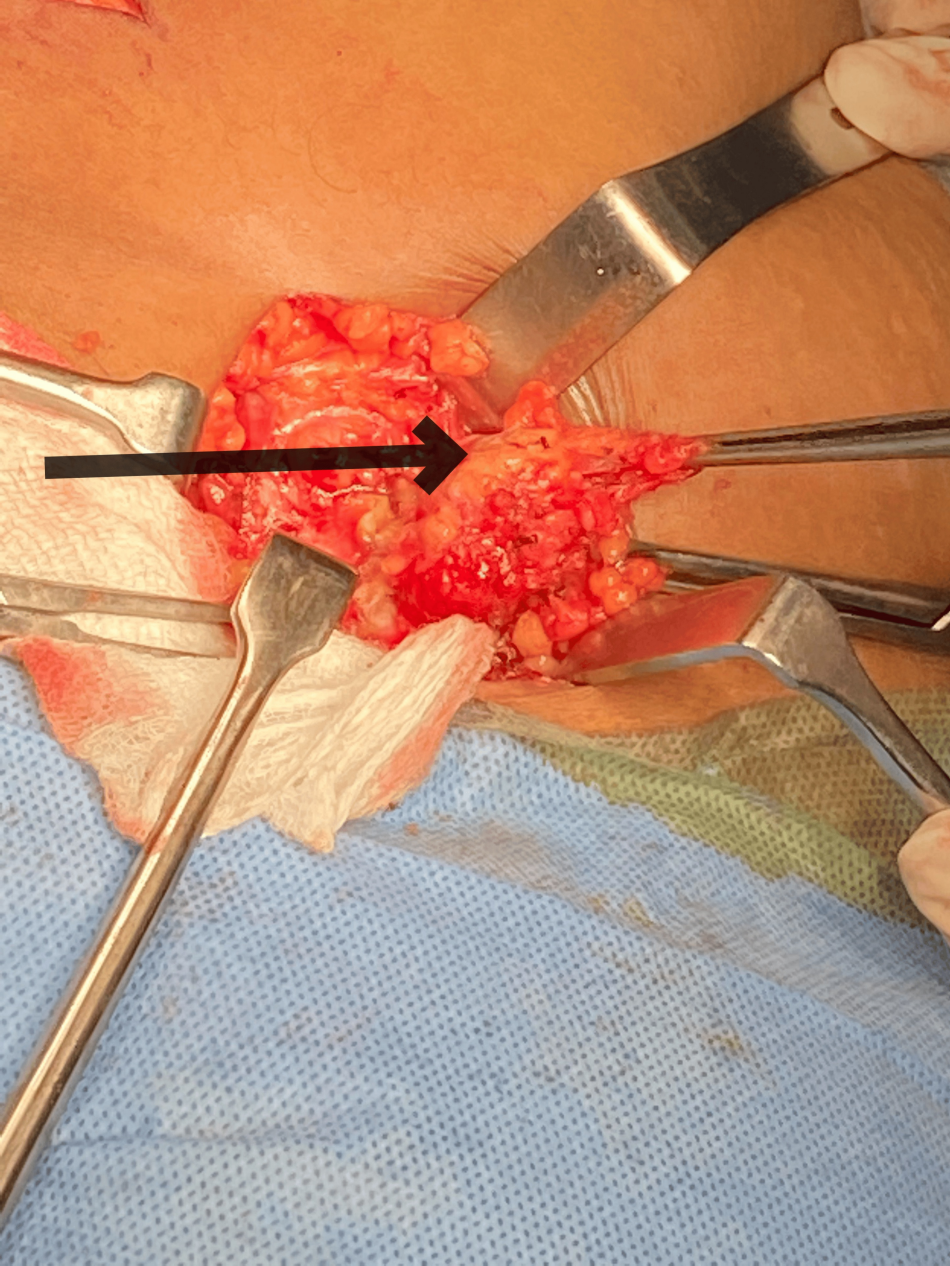
En-bloc dissection of the superficial right inguinal lymph nodes The black arrow shows a dissected superficial right inguinal lymph node.

**Figure 10:**
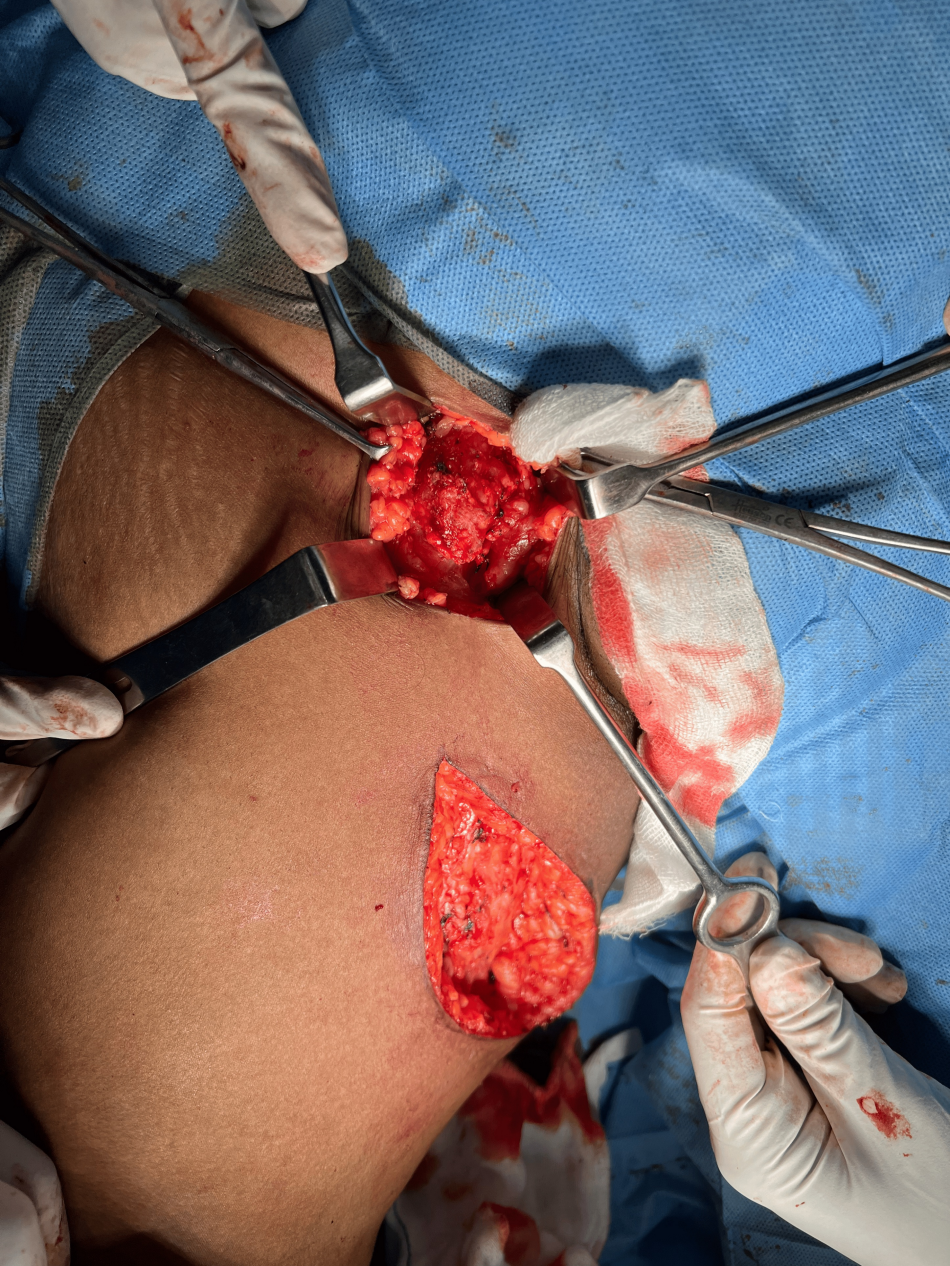
After the excision of the swelling along with the superficial right inguinal lymph nodes

Fine-needle aspiration cytology was performed on the swelling and lymph nodes, revealing atypical cells suggestive of malignancy and reactive lymph adenitis, respectively. The patient underwent a wide local excision with right inguinal en-bloc dissection under spinal anesthesia. Intra-operatively, the swelling was found to extend to the subcutaneous plane. After meticulous dissection, the great saphenous vein was ligated and divided, and the swelling was excised in toto, sparing the medial cutaneous nerve of the thigh (Figures 7-8). Subsequently, the right superficial inguinal nodes along the course of the great saphenous vein were identified and en-bloc dissection was performed (Figures 9-10).

The specimen and the inguinal nodes were sent for histopathology examination. The wound was carefully closed in layers after achieving hemostasis. The postoperative period was uneventful, with no sensory or motor deficits. The patient was followed up for 2 months, had no complaints, was symptom-free, and the wound healed well.

**Figure 11:**
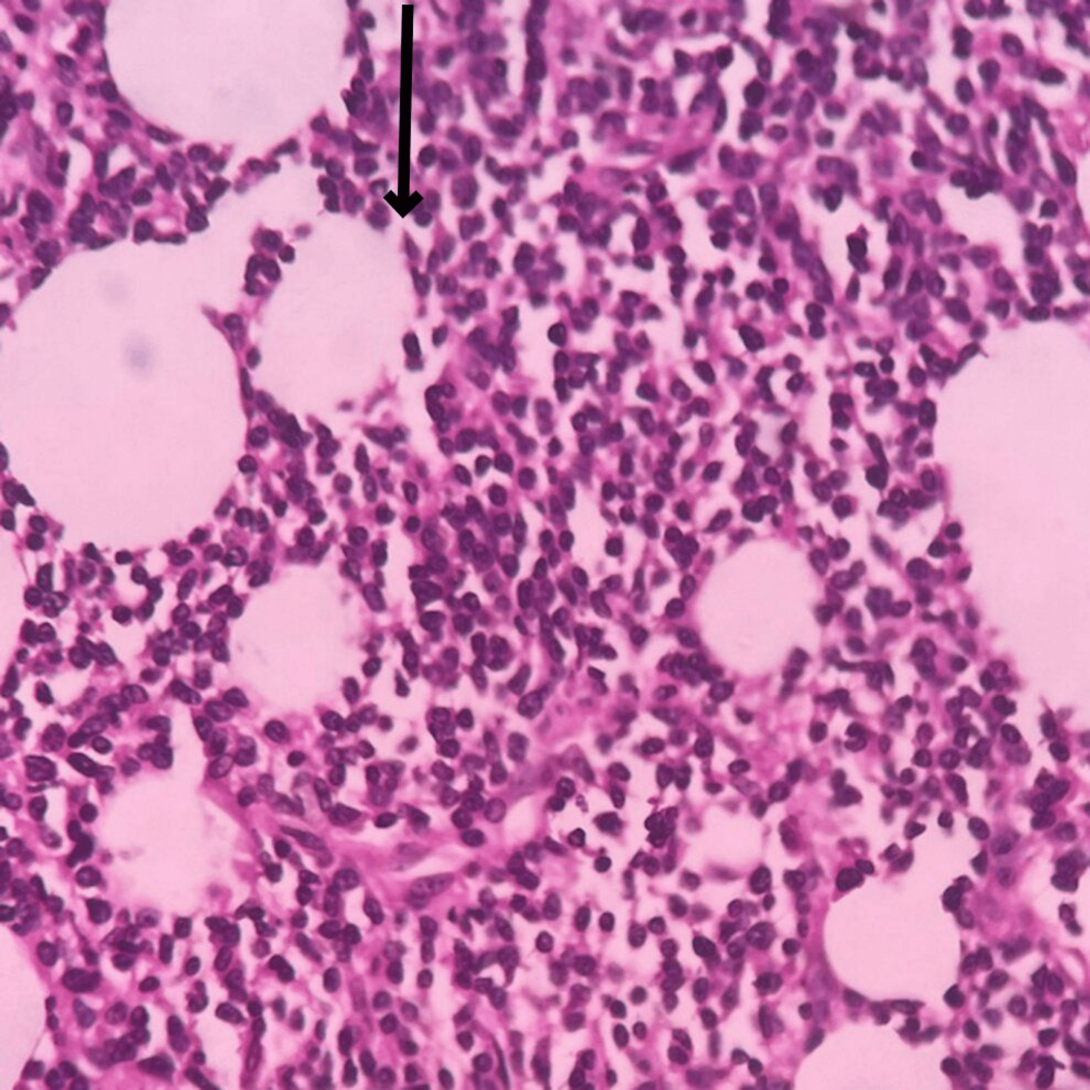
Microscopic slide showing the tumor extending into subcutaneous adipose tissue The black arrow shows the tumor extending into subcutaneous adipose tissue.

**Figure 12:**
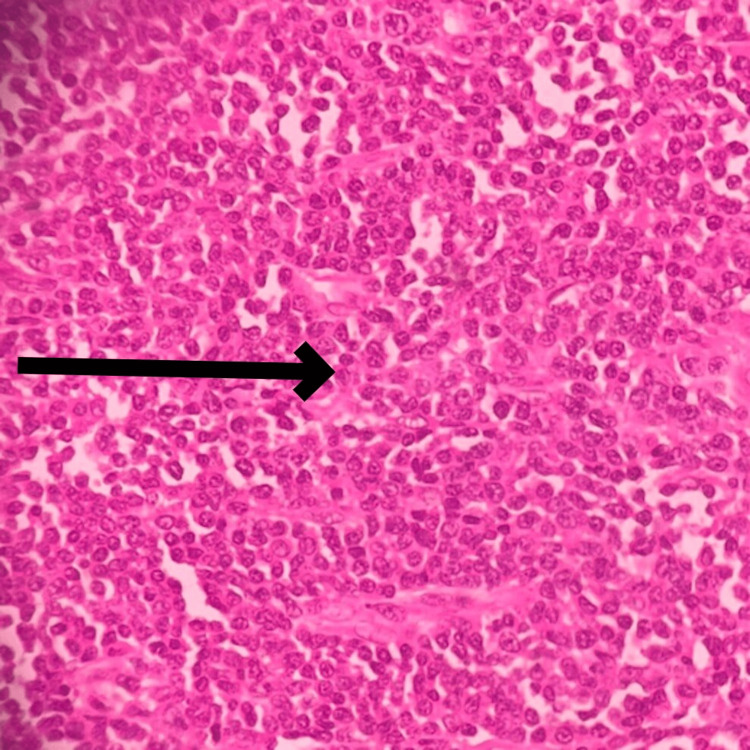
Microscopic slide showing the focal storiform arrangement of tumor cells The black arrow shows the focal storiform arrangement of tumor cells.

**Figure 13:**
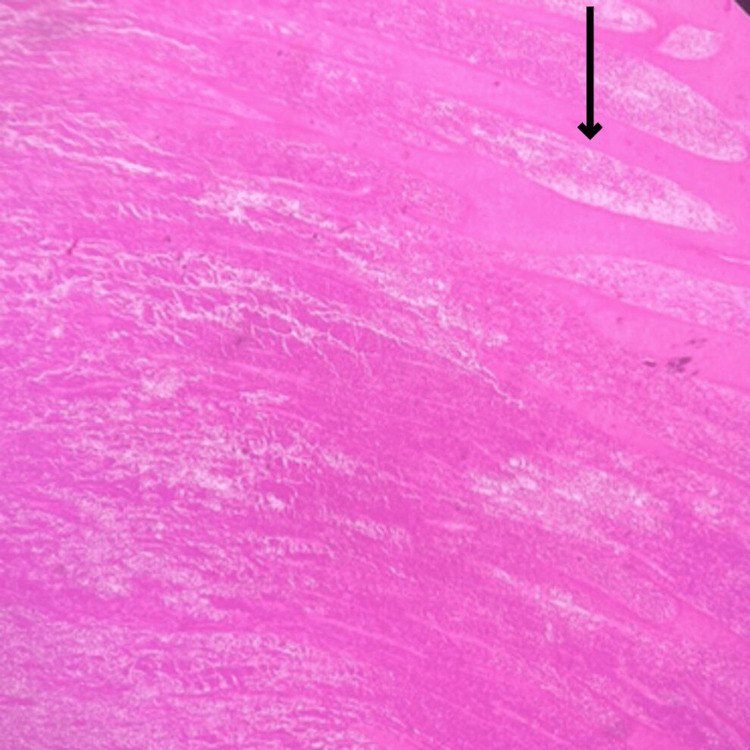
Microscopic slide showing the tumor cells have a spindle-shaped nucleus with eosinophilic cytoplasm The black arrow shows a spindle-shaped nucleus with eosinophilic cytoplasm.

Microscopic examination studied from the tumor properly showed sheets of atypical cells. Each cells have a predominantly round to oval vesicular nucleus with moderate eosinophilic cytoplasm with indistinct cell borders. Tumor was present in the dermis extending into subcutaneous adipose tissue (Figure 11). Individual adipocytes are seen trapped within sheets of tumor cells. The focal storiform arrangement of tumor cells was noted (Figure 12). In the deep dermis, tumor cells have spindle-shaped nuclei with eosinophilic cytoplasm (Figure 13).

**Figure 14:**
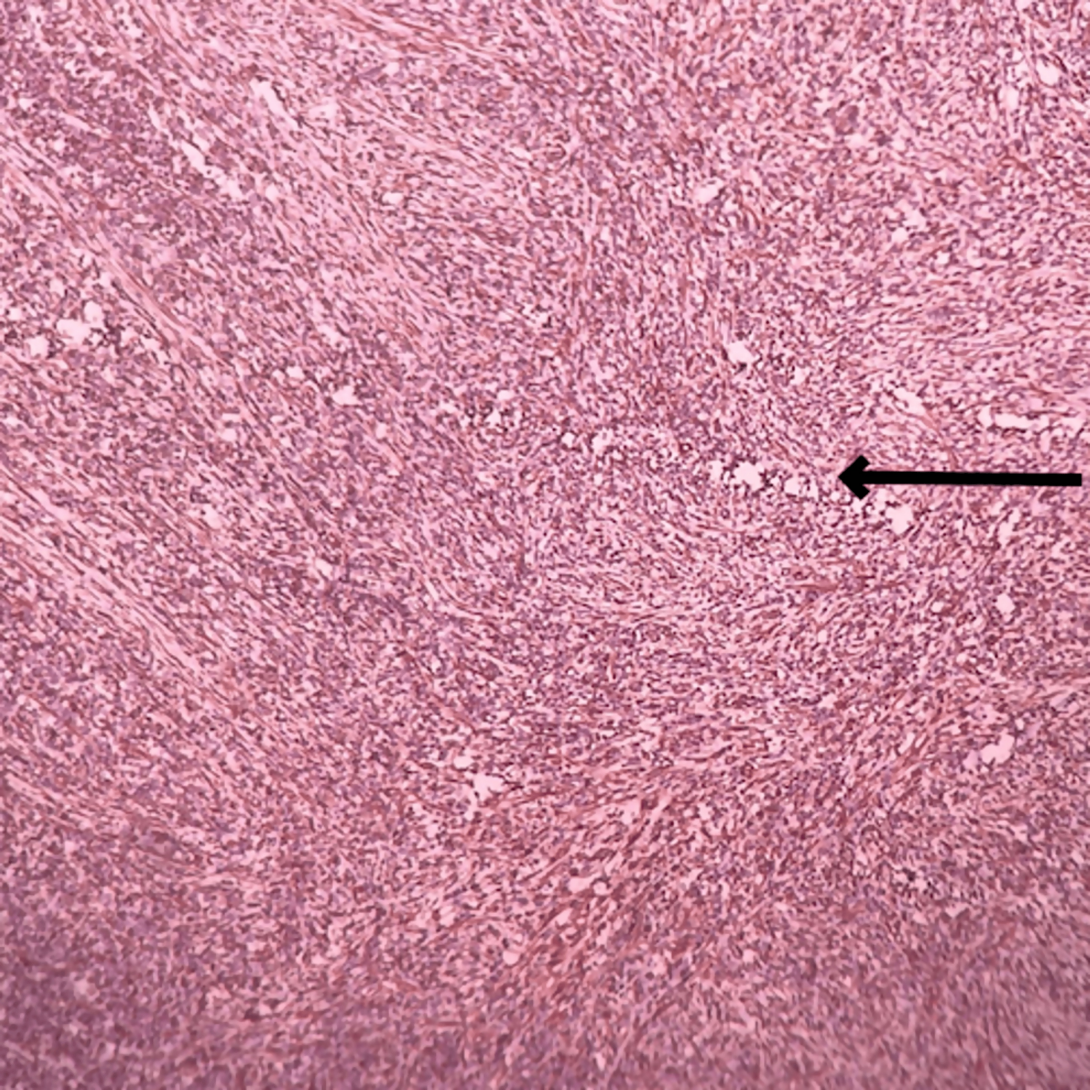
IHC marker showing diffuse cytoplasmic positivity for vimentin in tumor cells IHC: Immunohistochemistry The black arrow shows cytoplasmic positivity for vimentin in tumor cells.

**Figure 15:**
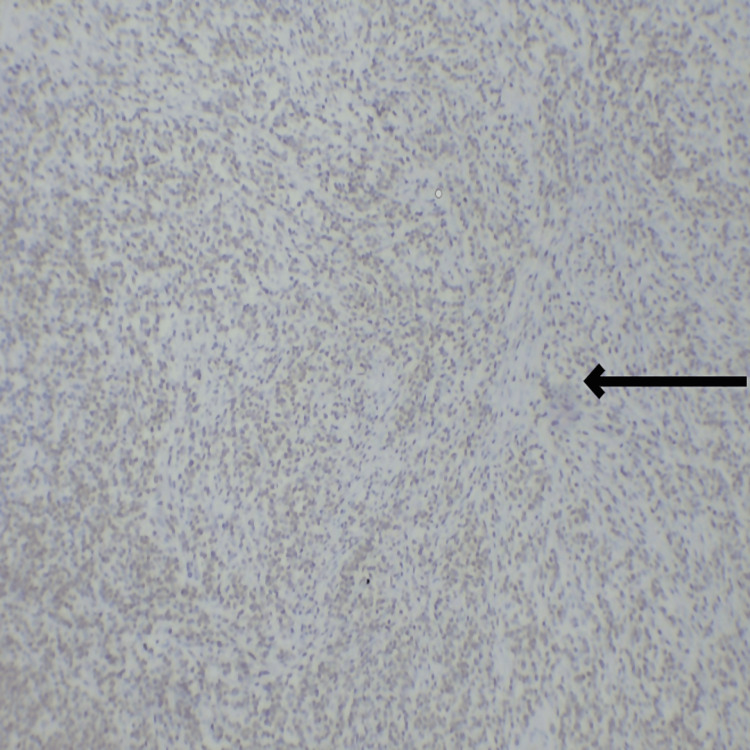
IHC marker showing weak membranous positivity for FLI-1 IHC: Immunohistochemistry The black arrow shows weak membranous positivity for FLI-1

The final impression was malignant undifferentiated soft-tissue sarcoma with predominantly round cell morphology. The tumor extends from the dermis and infiltrates into subcutaneous fat, with all surgically resected margins free of tumor. One of two inguinal lymph nodes shows involvement by a tumor with the same morphology. Further immune-histochemical markers were performed to confirm the cell origin, revealing diffuse cytoplasmic positivity for vimentin in tumor cells (Figure 14), membranous positivity for CD99 and CD31 in tumor cells, and weak positivity for FLI-1 (Figure 15), while testing negative for CD34, CD45, SMA, S100, NKX2.2, ERG, and MYOD1. This immunoprofile is suggestive of primitive neuroectodermal tumor (PNET)/EWS.

## Discussion

EWS is a highly metastatic type of sarcoma, ranking as the second most common primary malignant bone tumor, primarily affecting adolescents [4]. Although in our case it was a 44 years old female. EWS family tumors are characterized by a non-random chromosomal translocation that generates fusion genes encoding abnormal transcription factors. The t(11;22)(q24;q12) translocation, found in 85% of cases, results in the formation of EWS-FLI-1. In contrast, the t(21;12)(22;12) translocation leads to the less common EWS-ERG fusion, present in 10% to 15% of cases [5,6]. PNET, these tumors are believed to originate from fetal neuroectodermal cells and exhibit small-round-cell morphology with varying degrees of neural, glial, and ependymal features [7]. Primitive neuroectoderm tumors are categorized into central and peripheral types based on their cell of origin and location [8].

According to the WHO classification of round cell tumors, diffuse round cell pattern is classified into EWS, PNET, Merkel cell carcinoma, embryonal rhabdomyosarcoma (ERMS), small cell carcinoma, lymphoma, and leukemic infiltrate. It is widely acknowledged that EWS and peripheral primitive neuroectodermal tumor (pPNET) represent a unified neoplastic process, as evidenced by their overlapping morphological features, including ultrastructure, immunophenotype, and a shared set of molecular genetic abnormalities [9,10]. This neoplasm is now termed EWS/pPNET, which is the preferred terminology according to the World Health Organization classification of soft tissue and bone tumors [11], or alternatively, the Ewing family of tumors. These tumors originate from unique mesenchymal stem cells capable of differentiating into osteogenic, adipogenic, or neurogenic lineages. They exhibit a spectrum of behavior ranging from indolent to highly invasive and metastatic [1, 12].

EWS commonly affects anatomical sites such as the pelvis, axial skeleton, and femur; however, it can arise in almost any bone or soft tissue. Typically, patients present with pain and swelling at the affected site [4]. Despite its propensity for local spread, EES usually exhibits a pseudocapsule, which gives it a well-defined appearance on CT scans or MRIs.

Several diagnostic approaches are employed for confirming EWS/PNET. The first approach involves examining tumor tissue under light microscopy, including immunohistochemistry. These tumors are composed of primitive-looking round cells with a high nucleus-to-cytoplasm ratio [8]. A variety of immunohistochemical markers are utilized in the study of EES. These markers encompass the CD99 antigen, known for its high sensitivity though lack of specificity, and FLI1, which offers greater specificity compared to CD99. Molecular genetic analysis involves fluorescence in situ hybridization, which is considered indispensable. Immunohistochemical staining typically reveals diffuse positivity for CD99 (MIC2), FLI-1, and vimentin [13]. Detection of the *MIC2* gene through CD99 staining strongly suggests EES. In our case, there is strong positivity for CD99, vimentin, and weak positivity for FLI-1.

Radical excision surgery is considered the gold standard of treatment. Margin-negative surgery is crucial as there is no potential cure in sarcoma patients without achieving it [8]. Postoperative chemotherapy improves overall survival rates and reduces the risk of tumor recurrence [14], using a combination of multiple agents to enhance response rates [15]. Initially, regimens included vincristine, cyclophosphamide, actinomycin D, and doxorubicin (VAcCD). EWS can be complicated by metastases, local recurrence, secondary malignancies, pathological fractures, and morbidities associated with surgery, radiation, and chemotherapy [16].

## Conclusions

EWS are highly malignant tumors characterized by small, round cells originating from the neuroectoderm of bone and soft tissue. The exact histogenesis remains uncertain, but it is likely that the tumor cells arise from primitive mesenchymal cells with the potential for limited neural differentiation. A biopsy is essential for a definitive diagnosis, with commonly utilized techniques being open biopsy or imaging-guided core biopsy. Immunohistochemical and genomic studies play a crucial role in achieving accurate diagnosis. Modern therapy for EES follows the standard approach for all sarcomas in the Ewing family, involving extensive surgical resection followed by chemotherapy for systemic disease control and radiation for local disease control. Prognosis in EWS varies based on factors such as tumor size and location, presence of metastasis, and response to treatment.
